# Qualitative exploration of the early experiences of opioid use disorder patients from private clinics after Russia’s invasion of Ukraine in five major cities in Ukraine

**DOI:** 10.3389/fpubh.2023.1238188

**Published:** 2023-12-12

**Authors:** Alyona Mazhnaya, Anna Meteliuk, Iryna Pykalo, Frederick L. Altice

**Affiliations:** ^1^School of Public Health, National University of Kyiv-Mohyla Academy, Kyiv, Ukraine; ^2^International Charitable Foundation “Alliance for Public Health”, Kyiv, Ukraine; ^3^Ukrainian Institute on Public Health Policy, Kyiv, Ukraine; ^4^Department of Medicine, Section of Infectious Diseases, Yale University School of Medicine, New Haven, CT, United States; ^5^Division of Epidemiology of Microbial Diseases, Yale School of Public Health, Yale University, New Haven, CT, United States

**Keywords:** opioid use disorder, methadone, medications for opioid use disorders, resilience, war, Ukraine, health emergency

## Abstract

**Introduction:**

Following the full-scale invasion of Ukraine by the Russian Federation on 24 February 2022, over 6,000 patients were at risk of potential disruptions in treatment with medications for opioid use disorder (MOUD) in Ukraine. Before 2022, privatized MOUD clinics had emerged, partly driven by restrictive governmental policies and practices in state-funded facilities. Nevertheless, scant information exists regarding their operation and the patient’s experiences, especially during crises. This study seeks to elucidate the initial lived experiences of patients utilizing private MOUD clinics, integrating these insights with an analysis of the responding health system during war.

**Methods:**

The findings are derived from 20 qualitative semi-structured interviews conducted between March and June 2022, engaging participants from five major Ukrainian cities: Kharkiv, Kyiv, Odesa, Poltava, and Zaporizhzhya. Employing a rapid analysis procedure, we examined the data through descriptive and analytical summaries aligned with the domains of the data collection instrument.

**Results:**

Emergent themes encompassed stress and uncertainty following the invasion’s onset, challenges accessing MOUD, and consequent perceptions concerning state-funded versus private clinics. The study identified disruptions in the operation of private MOUD clinics across most cities examined. Issues pertaining to MOUD medication availability were linked to dosage reductions at state-funded clinics or pharmacy medication shortages or closures. Despite varied experiences at different MOUD clinics and cities, most participants continued their treatment.

**Discussion:**

This qualitative exploration provides a perspective on lived experiences with MOUD treatment at private clinics amidst the initial months of the invasion, illuminating how the early days’ stress, access challenges, varied responses from private MOUD clinics, and precarious conditions informed or altered preferences regarding MOUD treatment options. Moreover, these findings corroborate previously documented efforts by myriad stakeholders to mitigate war-related disruptions to MOUD delivery. These insights contribute to the international understanding of health system navigation and resilience during major crises, offering valuable lessons for preparedness development.

## 1 Introduction

The onset of the full-scale invasion of Ukraine by the Russian Federation generated diminished access to healthcare services and medicines nationwide. The reduction in access is particularly pronounced for residents near the front lines, in areas under partial Ukrainian government control, and among the internally displaced populations (1–4). Since the invasion began on 24 February 2022, there has been an exponential rise in the number of internally displaced people (IDPs), as reported by the UN Refugee Agency (UNHCR). As of June 2023, over 7.7 million individuals have been displaced, constituting 17.5% of the population. These displacements further exacerbate the stress on healthcare access and services (5, 6).

When the Russian Federation launched a full-scale invasion, over 17,000 patients nationwide received methadone or buprenorphine as their medication for opioid use disorder (MOUD) (7). In a month of a full-scale war, over 2000 patients faced the risk of MOUD interruption: at least 57 governmental MOUD clinics throughout Ukraine either stopped operating, were destroyed by shelling, or ran out of medications. Considering the total number of MOUD patients (over 17,000 at state-operated facilities and thousands more at private clinics), over 6,000 patients were at risk of treatment interruption in Ukraine (7).

War invariably leads to the immediate displacement and dispersion of people, uprooting lives and scattering communities in every direction. Displacement (8), the forced movement of people from their usual environments, and related dispersion—the distribution of displaced persons to different locations—perpetually complicate and disrupt the provision and continuity of healthcare services, making access to essential medical care a significant challenge. This is especially true for most MOUD that must be taken daily, without interruption, or patients will experience symptoms of abstinence, psychological distress and suicidal ideation and may lead to substance use relapse, overdose and death (9), which is what happened when Russia illegally annexed Crimea and banned all MOUD (10, 11). The UNHCR stresses that in crisis settings, where life-or-death allocation decisions are made under the principle of maximizing the greatest good for the greatest number, there is a need for an inclusive, deliberative, consistent, transparent, and technically sound decision-making process (8, 12). Implementing such a framework proves challenging, especially in diverse and often poorly governed environments (12, 13).

Wars and other devastating circumstances often overwhelm health systems, yet the Ukrainian health system continues to function despite the ongoing major shock stemming from the ongoing full-scale invasion. According to WHO estimates, the country’s health system has demonstrated resilience, maintaining overall high access to certain health services (1–3). The September 2022 estimates indicate that 95% of Ukrainians reported receiving primary care services among those seeking care, while up to 90% had access to health services for chronic conditions (3). Nonetheless, individuals with substance use disorders (SUDs) are particularly vulnerable during crises followed by displacement and risk disruptions in access to critical services and treatment (14, 15).

War introduces significant challenges to medication access due to supply chain interruptions, the closure of health facilities, and the displacement of the healthcare workforce, leading to reduced availability and accessibility of essential medications (12–17). Furthermore, displaced individuals often face substantial barriers in accessing medications, stemming from legal, financial, and logistical issues. The lack of stable housing and proper documentation often aggravates these challenges (12–15, 17). The trauma and stress associated with experiences of war and displacement also exacerbate mental health needs, leading to an increased demand for related services and medications—resources often scarce in such settings (18, 19). Additionally, there is evidence that IDPs experience higher mortality rates and worse health outcomes compared to host communities and refugees, with social determinants of health and limited access to essential health services as key drivers (18).

According to governmental reports and published studies related to the first few months following the initial invasion, the main challenges in MOUD provision were either real or perceived depletion of MOUD medications and the risk of supply interruptions, disruptions in data flow about existing supplies, the risk of closing MOUD clinics due to military activities, the destruction of health facilities and infrastructure, the occupation of the territories, and the displacement of MOUD patients and MOUD providers (4, 14, 20). Furthermore, on the fourth day of the invasion, Ukrainian borders were closed for all adult men under 60, increasing stress as men remained while their families fled Ukraine. This closure significantly affected patients with MOUD, as only those with certain levels of disabilities were permitted to leave. In rare instances, when these men left, they were provided with their medical records, history of treatment with MOUD, and contact information for MOUD treatment clinics in their destination countries at their original MOUD treatment clinics to facilitate prompt resumption of MOUD treatment upon arrival. Despite these initiatives, evidence suggests that sustaining MOUD treatment encounters challenges (21).

One recent study reported on governmental efforts to sustain MOUD treatment during the first year of the war, including important legislative changes and the mobilization of efforts that safeguarded treatment for thousands of MOUD patients across Ukraine; however, it acknowledged that there are limited data about those receiving MOUD from private treatment clinics in Ukraine (15).

Starting at the end of 2016 with changes in the legislative document guiding MOUD delivery, private MOUD treatment clinics emerged in parallel and, to some extent, as a reaction to restrictive government policies and practices in narcological facilities (22, 23) and patients’ willingness to pay for services to avoid such restrictions (24). These private clinics emerged as a practical approach to counteracting the restrictive practices within governmental clinics and addressing the unmet demand in MOUD (25–27). However, scant information is available regarding the operation of private MOUD treatment clinics, and even less is known about their response to the crisis precipitated by the war aside from one case report describing clinics in Kharkiv collaborating with government clinics (28–30).

This research analyzes the initial experiences of MOUD patients from private clinics within the context of the health system’s response to the Russian invasion of Ukraine on 24 February 2022. Through a systematic examination, this analysis elucidates the connection between patients’ lived experiences and the healthcare system’s counteractive measures implemented in the early wake of the invasion.

## 2 Methods

This study draws upon qualitative, semi-structured interview data collected as part of a more expanded implementation science research endeavor to understand the lived experience of people receiving MOUD in private settings in Ukraine at the onset of 2022. Data were collected from participants residing before the onset of the full-scale invasion in five major cities in Ukraine: Kharkiv, Kyiv, Odesa, Poltava, and Zaporizhzhya, which are representative of Ukraine’s Eastern, Northern, Central, and Southern regions, respectively.

We conducted a total of 20 interviews with individuals receiving MOUD medication at private clinics before the full-scale invasion of Russia on 24 February 2022. We recruited participants via targeted sampling, facilitated through direct collaboration with partner non-governmental organizations (NGOs) providing services to MOUD patients and participant referrals. Our team engaged social workers from partner NGOs at MOUD treatment clinics in each selected city. We provided these workers with the inclusion criteria for potential respondents and the contacts of the interviewer (AM-2) in case the respondent wanted to reach out directly. Social workers also provided contacts for potential participants after prior agreement with them. When the interviewer approached potential respondents directly via phone, there were no refusals to participate. We provided incentives to social workers for the recruitment (~3 USD). During data collection, we purposefully recruited to ensure geographical representation, socioeconomic characteristics, and a diverse participant pool from various backgrounds and regions. Participants were compensated ~14 USD for their time.

Only adults 18 or older receiving MOUD at a private clinic before 24 February 2022 were eligible. Before the interview, each study participant received extensive information about the study. Eligible participants provided oral consent. Interviews were conducted and recorded during online calls using the preferred messenger application, at times convenient for the participants, and either in Ukrainian or Russian based on the participant’s preference. Video conferencing was not utilized. Each interview, lasting approximately 30 min (AM-2), was conducted by an experienced researcher with substantial experience in qualitative interviewing of MOUD clients. We collected the data between March and June 2022. The semi-structured interview guide included the following domains: (a) pathways to MOUD treatment; (b) MOUD experience in private clinics; (c) MOUD experience since the beginning of the war; (d) current experience with MOUD; (e) other observed experiences with MOUD; (f) knowledge of the availability of illegal drugs since the full-scale invasion; and (g) social support for MOUD treatment. All audio recordings have been de-personalized; pseudonyms have been assigned to each interview, and these pseudonyms are used in quotes. For this analysis, the quotes were translated into English and back-translated into the original language by two research team members independently to ensure the quote’s essence is not lost in translation.

This study was reviewed and approved by the Institutional Review Boards at the Ukrainian Institute on Public Health Policy (Submission id 2019-009-04).

The interviews were recorded, and data were analyzed directly from the audio recordings using descriptive summaries and the domains of the data collection instrument. Further data analysis focused on summarizing and comparing participants’ experiences from different cities and developing analytical summaries (31).

Analysis was performed following a rapid assessment procedure (RAP) strategy (32), which organizes and summarizes the data according to a pre-defined framework. The steps of data analysis were as follows: (1) The data analysis team (AM-1, AM-2, and IP) listened to two interviews and agreed on key terms for the categorization within the RAP matrix that addressed the study’s aims; (2) AM-1 developed the categorization matrix pilot-tested by the data analysis team using three interview records to finalize the matrix; (3) AM-1, AM-2, and IP listened to all interviews and summarized them according to the matrix, which resulted in three individual matrices summarizing all the interviews according to the categories; (4) The data analysis team met to discuss differences and commonalities in the individual matrices; and (5) AM-1 combined matrices following the discussion and noted any disagreement in summaries. Discrepancies were assessed by the analytical team and resolved through iterative discussions. The final matrix was used to compare experiences by city and map findings into analytical categories described in the results section alongside key healthcare system’s counteractive measures.

## 3 Results

### 3.1 Participants

Table 1 provides the sampling of MOUD participants from private clinics in five major cities (Kyiv = 6, Kharkiv = 7, Poltava = 3, Odesa = 3, Zaporizhzhia = 1), and Table 2 provides the demographic and clinical characteristics before and soon after the invasion. One participant was female, and the majority were in their 30s and had initiated injecting drugs over 10 years before being prescribed methadone (*n* = 15) at a median dose of approximately 130 mg (12 mg for those taking buprenorphine). Before the full-scale invasion, more than half (*N* = 13) were either receiving MOUD as prescriptions that they could purchase at a pharmacy or were provided with take-home dosing (*n* = 6) that allowed them to intermittently attend the clinic for services. Half of the sample subsequently transitioned to a state-funded MOUD clinic within their city or in another location.

**Table 1:** Type MOUD clinics participants reported attending before February 24, 2022 and immediately after invasion and during data collection.

City	Before 24 February 2022		During data collection	
	Private MOUD clinics	State-funded MOUD clinics	Private MOUD clinics	State-funded MOUD clinics
Kyiv	6	0	1	5
Kharkiv*	7	0	3	4
Poltava	3	0	3	0
Odesa**	3	1	3	1
Zaporizhzhia	1	0	0	1

*Four people moved to Lviv and one person moved to Poltava.

**One person attended both types of clinics before and after 24 February 2022.

**Table 2:** Demographic characteristics of study participants (*N* = 20).

Participant characteristics	*N* = 20
Sex	
Female	1
Male	19
Median years	37
Medication for opioid use disorder (time of interview)	
Methadone, oral tablet	15
Buprenorphine, sublingual tablet	5
Type of MOUD clinic before 24th February → (during data collection)	
State-funded	0 → (10)
Private	19 → (9)
Both	1 → (1)
Form of MOUD receipt before 24th February → (during data collection)	
Daily at the MOUD clinic	1 → (0)
Take-home dosing	6 → (7)
By prescription at a pharmacy	13 → (13)
Median years of injection before MOUD initiation, years	14
Median and duration on MOUD, years	4
Median medication dosages (mg) before 24th February	
Methadone	134
Buprenorphine	13
Had their MOUD medication dosage decreased after 24th February	
No	10
Yes	10

Themes of stress and uncertainty were prevalent in the initial days after the full-scale invasion. The navigation of access to MOUD, and perceptions and lived experiences regarding state-funded versus private clinics were central to the participants’ experiences.

### 3.2 Double stress and uncertainties

Many participants were caught off-guard by the onset of the full-scale invasion of the Russian army into Ukraine; however, some mentioned concerns before 24 February 2022 about potential closures of their MOUD clinic in the event of military intervention. One participant even inquired about the contingency plans of the MOUD clinic in the case of a full-scale invasion, receiving a response in jest that suggested the clinic would continue its services without interruption.

*I asked them, what will happen when the war starts? - "You will work, won't you?" That's what they say - "We will even deliver to the trenches." They even made jokes*.

Stepan, from Kharkiv, moved to the clinic in Lviv

Participants were further stressed by the memory of the closure of MOUD in the Autonomous Republic (AR) of Crimea shortly after the Russian Federation annexed the peninsula in 2013.

*Well, now, God forbid, so that it doesn't turn out like in Crimea, because I already wanted to go to Germany. Here are the old ones [patients]; they are terribly afraid of this... Because we have already gone through it and know how it is and what it is.*

Oleksandr, from Odesa, changed the clinic in the same city

The abrupt onset of the invasion resulted in participants having varying amounts of medication on hand, sufficient for between 1 and 10 days of take-home dosing. Initially, participants mostly sought information regarding the operational status of their MOUD clinics and attempted to secure medication supplies to last. Experiences varied by city: some participants received confirmation that their clinics would continue operating as usual, alleviating immediate concerns; other clinics distributed the maximum allowable amount of medication available to patients; however, others became unreachable. These three approaches exemplified private MOUD clinics’ strategies to navigate through uncertainty: optimistic anticipation, proactive preparation for adverse outcomes, or relinquishment of responsibilities toward their patients.

*Everyone thought that it was temporary. On the day of the full-scale invasion, the clinics were working. Some people thought it was going to be a short scare. In a week, it became apparent that it was for a long time*.

Stepan, from Kharkiv, moved to the clinic in Lviv

Following the initial shock, participants were confronted with decisions emerging from concerns about the continuity of MOUD at their respective clinics and within their cities. They expressed insecurity and confusion regarding the possibility and means of continuing their MOUD treatment.

*I don't understand what's next... I was starting to half the pills... I left Kharkiv. I stayed in one place. I had to wait... Kharkiv, Pesochinka, Kremenchuk, Znamianka, Lviv one place, Lviv another place*.

Mykolai, from Kharkiv, moved to the clinic in Lviv

Participants adopted various approaches depending on whether their respective clinics remained operational, whether staff could continue working, the availability of medication in pharmacies for prescription, and concerns for personal and loved ones’ safety. They either continued receiving medication at private MOUD clinics (as observed in Odesa and Poltava), transitioned to governmental clinics within the same city (as in Kyiv), relocated to another city to access private clinics (e.g., participants from Kharkiv moved to Poltava), or switched to governmental clinics in a different city (e.g., Poltava or Lviv). These diverse experiences highlighted the disparate circumstances surrounding MOUD programs nationwide and the healthcare system’s response. Furthermore, participants’ initial wartime experiences influenced their attitudes toward different MOUD options, either reinforcing preferences or prompting reconsidering of long-term MOUD plans.

*I will stay at the state one [after the war's end]... I am somehow more comfortable here. I don't want to go anywhere*.

Oksana, from Kharkiv, moved to the clinic in Lviv

### 3.3 Access

The theme of access to MOUD medication encompassed the operation of MOUD clinics, staff availability, and the accessibility of medications (methadone and buprenorphine). These critical factors shaped the initial experiences of MOUD patients in the five major cities.

#### 3.3.1 MOUD clinic operations

Participants from most cities included in our study experienced disruptions in the operation of private MOUD clinics. Consequently, they either transitioned to another private MOUD clinic—undergoing the entry procedures anew, including paperwork, consultation, and urine tests—or switched to a governmental clinic.

*Initially, I went to a private clinic to make some kind of stock. But, unfortunately, everything was closed everywhere, as if no one ever worked there. I went to a governmental clinic and received mine [MOUD]. At the same time, I was monitoring the situation with private clinics*.

Petro, from Kyiv, changed the clinic in the same city

*There was one more private clinic, which started to work three days after the war had begun. I went as a new patient; again, I went through the whole procedure, consultations with a narcologist, documents, and a test for drugs in the urine*.

Oleksandr, from Odesa, changed the clinic in the same city

Participants also noted various challenges, including the absence of doctors to issue prescriptions and the closure or relocation of private clinics, particularly in Kharkiv, Kyiv, and Odesa. Despite initial uncertainties, when participants could contact their respective private MOUD clinics, most nurses and MOUD doctors were accessible at all clinics.

Disruptions in the operation of private MOUD clinics were not uniform. A participant from Odesa recounted how their private MOUD clinic not only continued its operations, but the staff also provided reassurance regarding the availability of prescriptions. Moreover, the staff strived to adhere to the prescription schedule and initiated processes to provide prescriptions for 30 days, which was allowed by the Ministry of Health. In another city, Zaporizhzhia, a participant described interruptions in the operations of the MOUD clinic and the pharmacy, noting that the clinic opened only 1–2 days per week. When unable to obtain his medication, the participant resorted to purchasing over-the-counter medications from the pharmacy to alleviate withdrawal symptoms. Some participants noted improvements in the conditions of MOUD clinic operations, such as receiving take-home medications for extended periods—up to 30 days.

*Doctors reassured us that there would be no difficulties or troubles. They said adjusting to the higher duration [of medication stock] is necessary, and I'll be able to receive for a month*.

Volodymyr, from Odesa, did not change clinics

*The hospital [MOUD clinic] worked irregularly, once or twice a week. There was a problem with the medication; the pharmacy was closed, did or did not have the drug. Sometimes I was missing my doses. I was taking “Lyrica” to have no withdrawals.*

Andriy, from Zaporizhzhia, changed the clinic in the same city

When participants experienced changing MOUD clinics within the same city, most reported a relatively smooth transition, often receiving medication on the same day. Those who were also internally displaced experienced interruptions and delays in commencing medication at new clinics. While evacuating from Kharkiv, one participant rationed available medication until reaching a new clinic in Lviv. The journey took approximately 4 days, with an additional couple of days required to locate the clinic and secure medication.

*On February 24 I had [medication] for nine days. I understood that no one knew what happens next, so from the first day I started to take half of a pill less. I have left the Kharkiv on a 7^th^ day, the trip took me four days... To stop somewhere was possible only on the 13^th^ day. I managed to take longer what I reduced gradually, but I felt bad, to be honest.*

Stepan, from Kharkiv, moved to the clinic in Lviv

#### 3.3.2 Medication

Challenges regarding the availability of MOUD medications were primarily associated with reduced dosages at governmental clinics or the absence of medication in pharmacies. Participants who attended private clinics and obtained MOUD through pharmacy prescriptions encountered significant obstacles, as medicines were frequently unavailable or the pharmacies were closed.

*A few days before the war started, I received a prescription from my private provider and came to the pharmacy to get my buprenorphine. The pharmacy was closed for us - drug users, even though people were working inside and many drug users with prescriptions outside. The territorial defence was not letting us in. I came the following day and the day after and could not buy buprenorphine with my prescription.*

Sergiy, from Kyiv, change the clinic in the same city

Governmental clinics in Kyiv continued operations with available medication; however, due to limited medication availability, doses were capped at a daily maximum of 100 mg of methadone, similar to the situation reported in Odesa. Participants from Poltava described fewer disruptions to clinic operations but observed a change in the type of methadone available (with the methadone produced in Odesa being supplied instead of the one produced in Kharkiv). Furthermore, the availability of methadone in pharmacies varied depending on whether the pharmacies received their methadone supplies from Kharkiv or Odesa. Some pharmacies ceased selling methadone altogether. At one point, methadone was reportedly available at only one pharmacy in Odesa, albeit without shortages. A participant from Zaporizhzhya indicated that prior to 24 February 2022, only one pharmacy in the city accepted prescriptions from private MOUD clinics, and this pharmacy ceased operations following the initiation of the full-scale invasion.

*The doctor explained that we have a limit. We cannot take more than 100 mg per 24 h. ***…*** …Kharkiv’s methadone disappeared, and Odesa’s [methadone] was in only one pharmacy in town. Well, from all clinics [people] were going there, and there were enough [medication]. There was no such situation that someone arrived and there was nothing there.*

Volodymyr, from Odesa, did not change clinic

An increase in the duration of take-home dosages or prescriptions was perceived positively but brought unintended financial consequences for patients at private MOUD clinics. These clinics often strongly recommended, or sometimes mandated, 30-day prescriptions, requiring patients to purchase a month’s medication in a single transaction. For instance, one private clinic altered its prescription duration policy, establishing a minimum of 20 days while encouraging 30-day prescriptions; this represented a change from previous options, which allowed for 5- or 10-day prescriptions. One participant initially welcomed receiving a 30-day supply from a private MOUD clinic but eventually ran out of funds and transitioned to a governmental MOUD clinic. Another participant, despite continuing with the program, expressed dissatisfaction with the modified prescription duration policy.

*Initially, I could buy for 5 or 10 days when I entered the clinic. The war began, a small amount of time passed, and the clinic changed this policy, starting to sell for at least 20 days, but preferably for 30.*

Oleksandr, from Odesa, did not change clinics

### 3.4 Preference for state-funded versus private clinics

In addition to the variation of participants’ experiences by city, they diverged by whether private MOUD clinics offered take-home doses or prescriptions. These experiences, intertwined with participants’ previous preferences for a MOUD modality and their socioeconomic status, either strengthened their inclination toward private MOUD, enhanced the appeal of governmental MOUD clinics, or led to the contemplation of stopping MOUD entirely. According to participant reports, private MOUD clinics that offered take-home doses or actively liaised with pharmacies were better prepared to maintain MOUD provision in the initial phase of the full-scale war. Furthermore, participants felt reassured when private MOUD clinics could relay information about the availability and location of MOUD medications in pharmacies, reinforcing the perception that these private entities are client-focused. Notably, these observations were primarily reported by participants in Poltava, a city comparatively less impacted at the time of data collection.

*On February 24, I had some supply of methadone at home, but I called my clinic right away asking what is happening, whether they were working, if I could come earlier to pick up additional medication… they assured me they were working regularly, just like before the war.*

Bogdan, from Poltava, did not change the clinic

Participants who opted to continue with private MOUD clinics accepted alterations in clinic operations, including price increases, extensions in the minimum number of days for which a prescription could be provided, and caps on maximum dosing. In our study, participants from Kyiv indicated that post-24 February 2022, only state-funded MOUD clinics remained operational. A majority transitioned from private to state-funded clinics due to the lack of responsiveness from the private MOUD clinics. As a result, those who transitioned to government-run MOUD clinics—amid closures or unresponsiveness of private MOUD clinics or pharmacies that previously filled prescriptions—developed a preference for governmental clinics owing to more reliable medication access. Furthermore, participants regarded governmental MOUD clinics as more dependable and sustainable amid the conflict due to their perceived obligation to citizens. In contrast, private clinics were viewed as commercial entities susceptible to abrupt closure and bearing no responsibilities toward citizens.

*I would choose a governmental clinic over a private. Why? First of all, it looked more reliable once the war broke in. It is more sustainable and seems to have certain responsibilities for its patients. And private – this is just business, just money, and business can shut down anytime.*

Petro, from Kyiv changed the clinic in the same city

Simultaneously, other participants expressed concerns that governmental MOUD clinics might face closure, akin to the discontinuation of MOUD experienced by hundreds in the AR Crimea in 2014. Such concerns led some individuals to contemplate ceasing MOUD entirely as a strategy to navigate the uncertainty surrounding MOUD in the event of occupation.

*I am planning to gradually taper off OAT [MOUD] if possible, maybe by 5 mg…I do not want to depend on it, on the system… What if the program closes?*

Stepan, from Kharkiv, moved to the clinic in Lviv

## 4 Discussion

This study analyzed the lived experiences of individuals undergoing MOUD treatment at private clinics in Ukraine within the initial months following the full-scale invasion by the Russian Federation. It provided new insights into the interplay between the stress and unpredictability characteristics of the invasion’s early days, the experiences associated with accessing MOUD, the varied responses from MOUD private clinics, and the precarious circumstances in which individuals found themselves. These experiences either strengthened or altered preferences toward the private MOUD modality. Some reports suggest that challenges persisted for individuals who evacuated outside Ukraine (21).

Two predominant MOUD clinic options, which operate independently, have been described in Ukraine: the state-affiliated MOUD program, which was implemented and overseen through a network of state-funded healthcare facilities, and the less explored option of private MOUD programs, which were fee-based and operated by private clinicians who worked closely with pharmacies (15, 25). The principal distinctions between these options lie in the payment structure for services provided and their visibility and reporting to state authorities. Private MOUD programs allow participants to remain unregistered (13) and largely undocumented by healthcare or other governmental authorities (15, 25).

The variable experiences reported by clients at different locations and certain private MOUD clinics suggested that the private MOUD sector in Ukraine is not homogeneous in its operations, resilience capacity, guiding principles, or the challenges posed by the unique circumstances arising from the full-scale invasion. These disparities in experiences and operational models offered valuable insights that can significantly contribute to discerning the effects of shock on privately run essential health services. Using the results of this study, researchers and policymakers can derive an understanding and perspectives that can inform and enhance approaches to handling crises in future. Appreciating how different MOUD service delivery models respond to and operate under crisis conditions is crucial for developing robust, resilient, and effective SUD treatment systems that absorb and adapt to crises.

Health system responses to acute conflict are time- and context-specific (33). Within our study, the identified theme of access—derived from experiences around the functioning of MOUD clinics, staff availability, and the accessibility of MOUD medications—was also a principal focus of governmental oversight (14, 15, 20).

During the initial months of warfare, the national MOUD hotline witnessed a 3-fold increase in calls, and a third of these inquiries originated from MOUD patients who had previously obtained their medications from private MOUD clinics, seeking information on enrollment in governmental programs (34). Our results underscored that securing sustained access to MOUD among patients at private clinics emerged as a paramount concern across various cities. Governmental and partner initiatives aimed at providing and coordinating vital information for MOUD patients were implemented, employing various channels, including chatbots, official websites, and direct communication at MOUD clinics (14, 15, 34). This streamlined communication strategy likely guided some patients at private MOUD clinics, aiding their decision-making on whether to transition to different clinics within their cities or to relocate altogether. Experiences reported by participants still varied based on the city and its proximity to conflict, ranging from private MOUD clinics actively providing information and assurance regarding the continuation of operations to others displaying non-responsiveness or complete shutdown of facilities. Earlier reports also highlighted the capability of certain private MOUD clinics to collaborate effectively among themselves and their governmental counterparts to ensure the continuity of MOUD (30). This further underscored the importance of swift, coordinated, and effective communication responses at all levels to preserve access to crucial healthcare services amidst the challenges posed by crises and displacement.

When some private MOUD clinics ceased operations altogether, governmental clinics demonstrated resilience and adaptability, enrolling more patients and maintaining medication dispensation by introducing capped maximum dosing. This response was reported through the triangulation of surveillance data, reporting mechanisms, and surveys targeting patients and providers (15).

Legislative amendments were pivotal in sustaining MOUD provision for individuals with SUD at state-funded facilities, as documented in governmental and international agency reports and peer-reviewed studies (14, 15, 20). These alterations included the authorization of MOUD take-home dosages for up to 30 days, the permission for healthcare facilities operating under martial law to store a 3-month supply of medication, the regulation of MOUD distribution to regions with critical MOUD needs, and the establishment of mechanisms enabling regions and healthcare facilities to request necessary medication amounts. These adjustments, based on the needs of internally displaced persons rather than existing schedules, exemplified the health system’s adaptive response to the crisis. These efforts also assured access to MOUD during the transition between clinics for many of our participants.

Some efforts also positively impacted private MOUD clinics, notably regarding take-home dosing and 30-day prescriptions and the supply of MOUD medication to areas with low stocks. The 30-day take-home dosing policy, however, had unintended consequences when private MOUD clinics prioritized this option, leading to financial constraints for some participants.

These findings provide valuable insights into the importance of agile policy responses and legislative adjustments amidst such crises, emphasizing the importance of considering the needs of MOUD patients regardless of the type of clinic where people receive services. The absence of such policies may lead to discontinuity of care, which some recall from tragic outcomes in AR Crimea, where disruptions led to overdose, suicide, an increased risk of HIV, and death (10). It highlights the need for flexibility in policy implementation to accommodate the needs of displaced individuals while also bringing attention to the unintended consequences that policy changes may have on patients.

Our study’s key finding, despite variations in individual experiences by specific MOUD clinics and different city contexts, is that participants predominantly managed to continue their MOUD following the initial shock and uncertainty engendered by the onset of the invasion. This continuity in MOUD is likely attributed to the health system’s and individuals’ resilience and adaptive capacity. The health system responded through strategic legislative and logistical initiatives by stakeholders and effective coordination, and individuals were remarkably resilient and able to navigate the complexity of a rapidly changing environment. As the war persists, especially with barrages of missiles and assaults across the country and remote from occupied territories, people with substance use disorders are increasingly vulnerable to stress (35, 36), which was observed as harm reduction services were disrupted by the war, making it crucial for both private and state-funded MOUD clinics to screen for and treat comorbid psychiatric and substance use disorders. These indicate the necessary next steps in service delivery adjustments to maintain individual and public health.

Despite the many important findings, this study is not without limitations. First, this qualitative research, employing rapid data collection and analysis, sought to offer insights into the lived experiences, priorities, preferences, and behaviors of MOUD patients at private clinics during the initial phase following the initiation of a full-scale invasion by the Russian Federation. These insights may be useful in documenting, systematizing, informing policy, interventions, and communication strategies. Nevertheless, these data cannot reflect the experience of patients with other services and health system responses nor the full variability of experiences of MOUD patients at private clinics in Ukraine, especially since we did not interview MOUD patients who were unsuccessful in continuing their treatment. We included efforts to conduct targeted sampling across different cities and continued interviews until saturation (at which no additional unique insights emerged) was achieved, followed by rigorous analysis. Individuals who were successful in navigating the challenging MOUD terrain, however, were more willing to share their experiences—possibly with less urgent, pressing concerns—might have been more inclined to participate in this study. While the experiences of individuals receiving MOUD treatment from private facilities during a significant crisis are scant in existing literature, it is imperative to acknowledge that these accounts may not fully capture the experiences of individuals in occupied territories or regions engaged in active combat. Second, online interviews without video have limitations compared to the often-preferred face-to-face format, though ample literature supports such approaches, especially during the recent COVID-19 pandemic (37, 38). Data collectors during such interviews may struggle to foster a trusting and comfortable atmosphere due to the absence of non-verbal cues and direct personal interaction. These factors could potentially impede the development of rapport between the interviewer and the participant. Nonetheless, during a full-scale invasion and significant mass displacement, utilizing these interviews represented the most pragmatic and feasible methodology available, as it was minimally disruptive and non-threatening for participants. It was also safer for patients and interviewers, overcame geographic constraints during the war, and allowed participants more anonymity. Crucially, a qualified and proficient team member who has accumulated experience conducting online interviews since the onset of the COVID-19 pandemic administered the interviews. Given the context, the benefits of employing online interviews significantly counterbalance the inherent limitations of this data collection mode. Third, we collected data in the early months of a full-scale invasion, thus not capturing the long-term experiences of MOUD patients. This limitation presents a research opportunity to explore the trajectories of this vulnerable population in a protracted crisis. Fourth, the mode of data collection constrained the depth of exploration for various topics, leaving some pertinent aspects of the experiences insufficiently examined. Future research endeavors may benefit from probing how stress and uncertainty during crises might drive individuals to utilize non-prescribed opioids and other illicit substances. It is also vital to scrutinize the strategies individuals deploy to navigate stress and uncertainty during emergencies and understand the availability and efficacy of formal and informal crisis intervention mechanisms accessible to individuals in these periods.

This study enriches the existing body of knowledge regarding the experience of substantial crises that disrupt essential public health and healthcare services, offering insights that bear global relevance. It underscores managing uncertainty and access as pivotal elements of health system resilience, applicable to both the public and private sectors. Moreover, the accounts provided by our participants corroborate previous reports (14, 15, 20), illustrating the concerted efforts by stakeholders at various levels to mitigate the disruption wrought by the war on MOUD delivery. These findings offer valuable lessons for international audiences, highlighting adaptive strategies and responses integral to sustaining critical healthcare services amidst crises.

## Data availability statement

The datasets presented in this article are not readily available because the dataset consists of audio records of online interviews with patients receiving MOUD. We opt not to make this dataset available to protect the confidentiality of participants. Upon request, however, we may provide summaries of analytic categories and transcripts (but not audio-recordings themselves). Requests to access the datasets should be directed to hmazhnaya@gmail.com.

## Ethics statement

This study was reviewed and approved by the Institutional Review Boards at the Ukrainian Institute on Public Health Policy (Submission id 2019-009-04). The studies were conducted in accordance with the local legislation and institutional requirements. The ethics committee/institutional review board waived the requirement of written informed consent for participation from the participants or the participants’ legal guardians/next of kin because the data collection was conducted through online interviews, written informed consent would have been the only document identifying our subjects.

## Author contributions

AlM: conceptualization, methodology, formal analysis, validation, data curation, writing – original draft, and writing – review and editing. AnM: project administration, resources, writing – review and editing, and supervision. IP: project administration, and writing – review and editing. FA: writing – review and editing, supervision, and funding acquisition. All authors contributed to the article and approved the submitted version.
